# Books Received

**DOI:** 10.1093/medlaw/fww033

**Published:** 2016-12-22

If you are interested in writing a review of one of the books listed below please contact the Book Reviews Editor, Dr Sara Fovargue: s.fovargue@lancaster.ac.uk. Reviews are usually about 2,000 words long, but shorter reviews will be considered with the agreement of the Reviews Editor.

P Balen (ed), *Clinical Negligence* 2nd ed (Jordan Publishing, 2015).

D Bowen Matthew, *Just Medicine: A Cure for Racial Inequality in American Health Care* (New York University Press, 2016).

British Medical Association and the Law Society, A Ruck Keene (ed), *Assessment of Mental Capacity: A Practical Guide for Doctors and Lawyers* 4th ed (British Medical Association, 2015).

E Cloatre, M Pickersgill (eds), *Knowledge, Technology and Law* (Routledge, 2015).

LC Edozien, *Self-Determination in Health Care: A Property Approach to the Protection of Patients’ Rights* (Ashgate, 2015).

I Glenn Cohen, *Patients with Passports: Medical Tourism, Law, and Ethics* (Oxford University Press, 2015).

IO Iyioha, RN Nwabueze, *Comparative Health Law and Policy: Critical Perspectives on Nigerian and Global Health Law* (Ashgate, 2015).

ED Kinney, *The Affordable Care Act and Medicare in Comparative Context* (Cambridge University Press, 2015).

The Law Society, *Deprivation of Liberty: Collected Guidance* (Law Society, 2016).

J-Y Lee, *A Human Rights Framework for Intellectual Property, Innovation and Access to Medicines* (Ashgate, 2015).

N Lunt, D Horsfall, J Hanefeld (eds), *Handbook on Medical Tourism and Patient Mobility* (Edward Elgar, 2015).

K Sideri, *Bioproperty, Biomedicine and Deliberative Governance: Patents as Discourse on Life* (Ashgate, 2015).

MK Smith, *Saviour Siblings and the Regulation of Assisted Reproductive Technology: Harm, Ethics and Law* (Ashgate, 2015).

